# A Robust Observer-Based Control Strategy for n-DOF Uncertain Robot Manipulators with Fixed-Time Stability

**DOI:** 10.3390/s21217084

**Published:** 2021-10-26

**Authors:** Anh Tuan Vo, Thanh Nguyen Truong, Hee-Jun Kang, Mien Van

**Affiliations:** 1 Department of Electrical, Electronic and Computer Engineering, University of Ulsan, Ulsan 44610, Korea; voanhtuan2204@gmail.com (A.T.V.); thanhnguyen151095@gmail.com (T.N.T.); 2 School of Electronics, Electrical Engineering and Computer Science, Queen’s University Belfast, Belfast BT7 1NN, UK; m.van@qub.ac.uk

**Keywords:** uniform robust exact differentiator, nonsingular terminal sliding mode control, fixed-time control, robot manipulators

## Abstract

In this paper, a robust observer-based control strategy for n-DOF uncertain robot manipulators with fixed-time stability was developed. The novel fixed-time nonsingular sliding mode surface enables control errors to converge to the equilibrium point quickly within fixed time without singularity. The development of the novel fixed-time disturbance observer based on a uniform robust exact differentiator also allows uncertain terms and exterior disturbances to be proactively addressed. The designed observer can accurately approximate uncertain terms within a fixed time and contribute to significant chattering reduction in the traditional sliding mode control. A robust observer-based control strategy was formulated, according to a combination of the fixed-time nonsingular terminal sliding mode control method and the designed observer, to yield global fixed time stability for n-DOF uncertain robot manipulators. The proposed controller proved definitively that it was able to obtain global stabilization in fixed time. The approximation capability of the proposed observer, the convergence of the proposed sliding surface, and the effectiveness of the proposed control strategy in fixed time were fully confirmed by simulation performance on an industrial robot manipulator.

## 1. Introduction

Over the past decade, robot manipulators have drawn major attention in academia and across industries. The potential applications of the robot are wide-ranging. Currently, robots can be found working in many fields, such as deburring, welding, automotive industry, bomb detection, ocean exploration, polishing, surgery, agriculture, and so on. In these applications, robots run in components of physical interaction with the working environment. It is well-known that nonlinearities and uncertain dynamics occur widely in robot manipulators, and include unstructured uncertainties and structured uncertainties. Furthermore, exterior disturbances, payload variations, and sensor noise cannot be prevented. These issues can reduce the control performance, stability, safety, and reliability of robots. Hence, more attention should be focused on proposing efficient controllers with robust anti-uncertainty ability, fast convergence rates, small overshoot, and high accuracy. To test the effectiveness of the control methods, motion tracking control of robot manipulators is a popular topic in engineering and science.

In recent years, several different control algorithms were proposed for robot manipulators. They mostly included linear control strategies and nonlinear strategies, such as the proportional–integral–derivative (PID) control [1,2], linear quadratic regulator (LQR) [3], computed torque control (CTC) [4], backstepping control [5], model predictive control (MPC) [6], and sliding mode control (SMC) [7,8], which were integrated into the motion control of the robot manipulators. Linear control strategies are strictly limited to a limited domain, leading to difficulties in scaling to most real-time applications. Nonlinear control strategies could improve stability and expand the operation domain. Therefore, nonlinear control strategies frequently attract more attention than linear control strategies in controlling robotic manipulators. However, most of the mentioned nonlinear methods are highly sensitive to uncertainty terms, or require precise model parameters. These intrinsic weaknesses can be handled by applying SMC.

SMC is not sensitive towards external disturbances and parametric uncertainties. It can effectively compensate for the effects of uncertain terms. Therefore, SMC has been widely implemented in real robot applications. Unfortunately, SMC only provides exponential convergence, while the control inputs involve undesirable oscillation. For the exponential convergence, the trajectory of the control errors only reaches zero once time goes to infinity. To achieve higher performance, faster convergence performance is required to match real systems. Hence, asymptotic convergence seems to be unsuitable for applications requiring high accuracy. Furthermore, oscillation also known as chattering, leads to undesirable mechanical stress on actuators and the structure of robot manipulators [9].

A great deal of effort has been devoted to the finite-time convergence guarantee of the system states. The control methods that could provide finite-time stability include the higher-order sliding mode control (HOSMC) [10,11], the terminal SMC (TSMC) [12,13], the nonsingular TSMC (NTSMC) [14,15], fast TSMC (FTSMC) [16,17], global FTSMC (GFTSMC) [18,19], the nonsingular fast TSMC (NFTSMC) [20,21], and the finite-time TSMC (FnTSMC) [22,23]. HOSMC is capable of providing finite-time stability, and chattering reduction can also be achieved by regularization of switching functions and by considering (virtual) actor dynamics as input low-pass filters. Most of the mentioned TSMC-based methods performed so far have not rigorously solved problems such as chattering or slow convergence in finite time control when the initial starting point of the system’s trajectories has a large value. In addition, these methods involve a trade-off between chattering behavior and control performance. Due to dependence on initial conditions, convergence time rises unlimitedly once those conditions go to infinity, in the theory of finite-time controllers. To minimize that dependency, fixed-time control methods were proposed [24,25]. The main advantage of fixed-time controllers is that the convergence time can be pre-computed by setting appropriate design constants, which are bounded. These controllers often exhibit excellent performance and powerful disturbance cancellation. Therefore, they are increasingly applicable to robotic systems [26,27].

It is well-known that the existence of uncertain terms in the robotic system is inevitable. Therefore, it is necessary to enhance the robustness and durability of the controllers under the influence of uncertain terms. In the literature, numerous observer-based control strategies were proposed. For example, an observer-based control strategy was proposed for fault-tolerant control (FTC) of the robot manipulators [28], and the extended observer-based synchronous SMC scheme for FTC of the robot manipulators was developed [29]. However, these observer-based controls only ensure asymptotic stability. In addition, there are a few more proposed observers, such as the high-gain observer (HGO) [30] and the third-order sliding mode observer (TOSMO) [31]. While HGO only ensures asymptotic convergence in the study [30], the proof of finite-time convergence has not been fully yielded in the study [31]. The Kalman filter (KF) is one of the most extensively applied approaches for monitoring and estimation. KF’s advantages include observability, simplicity, controllability, smoothing, optimality, and robustness [32,33,34]. Unfortunately, using KF for nonlinear systems can face many difficulties and problems. To apply the traditional linear Kalman filter to nonlinear systems, the most common method is to employ an Extended Kalman Filter (EKF), which simply linearizes all nonlinear models. As mentioned above, linear approaches are strictly limited to a limited domain, leading to difficulties in scaling to most real-time applications. Therefore, the application of KF to the design of robot control seems to be unsuitable.

In recent years, to further enhance the accuracy and speed of perturbation estimates, SMC methods based on finite-time disturbance observers (FnDOs) [35,36] or fixed-time disturbance observers (FxDOs) [26,27] were proposed. In the studies [35,36], because the convergence time of FnDOs relies on the initial conditions, it increased indefinitely as those conditions went to infinity. In the study [26], the authors developed an FxDO to estimate uncertain terms of nonlinear systems. However, FxDO will not effectively estimate perturbation when the data from the accelerometer is obtained to be degraded. In the works [37,38], robust exact differentiators combined with SMC were introduced to improve performance, including estimation accuracy and robustness against measurement errors and chattering phenomena reduction. Nevertheless, once the norm of the initial conditions rose unlimitedly, the convergence time of the observers/differentiators tended toward infinity. It should be noted that the observer’s convergence property in a fixed time is important for separation-like properties in robot manipulators. It implies that the observer’s estimation errors reach zero before the real trajectories of the robot have flowed to infinity. To achieve both estimation accuracy and robustness in fixed time, and to remove the dependence of the initial conditions, a uniform robust exact differentiator (URED) was proposed [39]. An arbitrary order differentiator was further developed in the study [40].

Most of the observer-based control strategies introduced so far guarantee that the estimation errors or the tracking error will approach to equilibrium point within finite time. Numerous methods achieve asymptotic convergence of both types of the mentioned errors. Some observers/differentiators only focus on estimating the unmeasurable states, and ignore the effect of uncertainty or disturbance under time-varying impacts on the robot manipulators. Moreover, because chattering is a key weakness of the SMC methods, we also need to focus on this problem.

Based on the stated goal, our paper developed an observer-based control algorithm for n-DOF robot manipulators under the existence of uncertain terms. This was developed with the important contributions below, which facilitated the proposed work for real-time implementation.

- The novel fixed-time nonsingular terminal sliding mode (FxNTSM) surface was proposed to quickly obtain a fixed-time convergence of the control errors without singularity.
- To proactively deal with uncertain terms and exterior disturbances, the FxDO was developed based on a URED. The designed FxDO accurately approximated uncertain terms within a fixed time and contributed to significantly reduced chattering in the traditional SMC. In addition, the proposed FxDO removed the requirements for measuring acceleration, as presented in high-order sliding mode (HOSM) observers [26,41].
- The proposed controller had a simple design suitable for extension to actual robots. It was formed according to a combination of the fixed-time nonsingular terminal sliding mode control (FxNTSMC) method and the designed FxDO, to offer global fixed-time stability for robot manipulators. The convergence time was able to be pre-computed by setting appropriate design constants, which were bounded.
- The proposed controller obtained high tracking accuracy, small overshoot, chattering reduction, robust anti-uncertainty ability, and fast convergence of both the tracking errors and the estimation errors within fixed time.
- The proposed FxNTSMC proved definitively that it was able to obtain global stability in fixed time using the Lyapunov criteria.

The arrangement of the article is presented as follows. Following the introduction, the assumptions, basic definitions, lemmas, and problem formulations are described in Section 2. The control design in Section 3 describes a novel FxNTSM surface, a novel FxDO based on a URED, and a novel FxNTSMC strategy. In Section 4, a 3-DOF industrial robot system simulated under the existence of uncertain terms is used to investigate the control performance of the suggested control strategy. Finally, notable conclusions from the proposed theory and simulation results are summarized in Section 5.

To assist readers, the list of notations and nomenclature is given in Table 1.

## 2. Problem Statement, Basic Definitions, and Assumptions

### 2.1. Description of Robot Manipulators’ Dynamic Model

A description of an n-DOF uncertain robot manipulators’ dynamic model is presented along with disturbance, as follows:

(1) $$M\left(p\right)\ddot{p}+C\left(p,\dot{p}\right)\dot{p}+G\left(p\right)+f_{r}\left(\dot{p}\right)=\tau -\tau_{d}\left(t\right).$$

In fact, the dynamics of the robot involve uncertain terms with high nonlinearity, such as wear, Coulomb friction, varying payload, etc. For complete consideration, the terms of dynamical uncertainty are described as follows: $M\left(p\right)=\hat{M}\left(p\right)+\Delta M\left(p\right)$, $C\left(p,\dot{p}\right)=\hat{C}\left(p,\dot{p}\right)+\Delta C\left(p,\dot{p}\right)$, and $G\left(p\right)=\hat{G}\left(p\right)+\Delta G\left(p\right)$.

Set $v={\left[v_{1}^{T},v_{2}^{T}\right]}^{T}={\left[p^{T},{\dot{p}}^{T}\right]}^{T}$ and u=τ; accordingly, the dynamic model of the robot (1) is depicted in state space by:

(2) $$\{\begin{array}{l} {\dot{v}}_{1}=v_{2}\\ {\dot{v}}_{2}=Ζ\left(v\right)u+A\left(v\right)+\delta \left(v,\Delta ,\tau_{d}\right) \end{array},$$

where $A\left(v\right)=-{\hat{M}}^{-1}\left(p\right)\left(\hat{C}\left(p,\dot{p}\right)\dot{p}+\hat{G}\left(p\right)\right)$, $Ζ\left(v\right)={\hat{M}}^{-1}\left(p\right)$, and $\delta \left(v,\Delta ,\tau_{d}\right)=-{\hat{M}}^{-1}\left(p\right)\left(f_{r}\left(\dot{p}\right)+\Delta M\left(p\right)\ddot{p}+\Delta C\left(p,\dot{p}\right)\dot{p}+\Delta G\left(p\right)+\tau_{d}\right)$.

Define $e_{1}≜p-p_{d}$, $e_{2}≜\dot{p}-{\dot{p}}_{d}$, and ${\dot{e}}_{2}≜\ddot{p}-{\ddot{p}}_{d}$, so, the system (2) can be formulated with the form involved the control errors:

(3) $$\{\begin{array}{l} {\dot{e}}_{1}=e_{2}\\ {\dot{e}}_{2}=Ζ\left(v\right)u+ℋ\left(𝒗\right)+\delta \left(v,\Delta ,\tau_{d}\right) \end{array},$$

where $e={\left[\begin{array}{cc} e_{1}^{T} & e_{2}^{T} \end{array}\right]}^{T}\in R^{2n}$ indicates vector of control errors and $ℋ\left(v\right)=-{\hat{M}}^{-1}\left(p\right)\left(\hat{C}\left(p,\dot{p}\right)\dot{p}+\hat{G}\left(p\right)\right)-{\ddot{p}}_{d}$ represents the smooth nonlinear function.

### 2.2. Basic Definitions and Assumptions

Lemmas, definitions, and assumptions are necessary for the design procedure of the proposed controller and proof of convergence and stability in finite time or fixed time.

The sign(·) function is described with the following expression:

$$\mathrm{sign}\left(v\right)=\{\begin{array}{l} 1\;if\;v>0\\ 0\;\mathrm{if}\;v=0\\ -1\;\mathrm{otherwise} \end{array}.$$

It can be clearly confirmed that as φ≥0

$$\{\begin{array}{c} \mathrm{sig}{\left(v\right)}^{\varphi }={\left|v\right|}^{\varphi }\mathrm{sign}\left(v\right)\\ \frac{d}{dt}\mathrm{sig}{\left(v\right)}^{\varphi }=\varphi {\left|v\right|}^{\varphi -1}\dot{v} \end{array}.$$

**Assumption** **1:** *The system states of Equation (1) for controls are bounded for all time.*

**Assumption** **2.** *Assume that the lumped unknown uncertainty at each joint is bounded by:*

(4) $$\left|\delta_{i}\left(v,\Delta ,\tau_{d}\right)\right|<\varrho_{i},$$

*where* $\varrho_{i}$* is a positive constant.*

**Assumption** **3** ([42]). *Assume that the first derivative of the lumped unknown uncertainty at each joint is bounded by:*

(5) $$\left|{\dot{\delta }}_{i}\left(v,\Delta ,\tau_{d}\right)\right|<{\bar{\varrho }}_{i},$$

*where* ${\bar{\varrho }}_{i}$ *is a positive constant.*

Let us consider autonomous system as follows:

(6) $$\dot{v}\left(t\right)=f\left(v\left(t\right)\right),v\left(0\right)=v_{0},$$

where $v\in R^{n}$ and f: $R^{n}\to R^{n}$ is a nonlinear function. Let us assume that the origin is an equilibrium point of Equation (6).

**Definition** **1** ([27])**.** *The equilibrium point of Equation (6) is considered to be a finite-time stable equilibrium if the origin is Lyapunov stable, and any solution* v(t) *starting from* $v_{o}$ *satisfies* $\underset{v\to \infty }{lim}v\left(t,v_{0}\right)=0$ *for all* $t\geq T\left(v_{0}\right)$ *, where* T *:* $R^{n}\to R^{+}$ *is called the settling time function.*

**Definition** **2** ([27])**.** *The equilibrium point of Equation (6) is considered to be a fixed-time stable equilibrium if it is globally finite-time stable and its bounded convergence time* $T\left(v_{0}\right)<T_{max}$ *, where* $T_{max}>0$ *is a positive number.*

**Lemma** **1** ([43])**.** *Let us consider the scalar differential equation, as follows:*

(7) $$\dot{ℒ}\left(v\right)=-\kappa \mathrm{sig}{\left(ℒ\left(v\right)\right)}^{\alpha },$$

*where* κ>0 *,* 0<α<1*. Then, the origin is a finite-time-stable equilibrium of Equation (7), and the settling time T is satisfied by the following inequality:*

(8) $$T_{1}\leq \frac{1}{\kappa \left(1-\alpha \right)}{ℒ}^{1-\alpha }\left(v\left(0\right)\right).$$

**Lemma** **2** ([27]). *Let us consider a scalar differential equation, as follows:*

(9) $$\dot{ℒ}\left(v\right)=-Z_{1}\mathrm{sig}{\left(ℒ\left(v\right)\right)}^{𝒿}-Z_{2}\mathrm{sig}{\left(ℒ\left(v\right)\right)}^{𝓂},$$

*where* $Z_{1},Z_{2}>0$ *,* 𝒿>1 *, and* 0<𝓂<1.

*Then, the system (7) is globally fixed-time stable and the convergence time* $T_{2}$ *is given by:*

(10) $$T_{2}<\frac{1}{Z_{1}}\frac{1}{𝒿-1}+\frac{1}{Z_{2}}\frac{1}{1-𝓂}.$$

## 3. Robust Observer-Based Control Strategy for n-DOF Uncertain Robot Manipulators with Fixed-Time Stability

In this section, a robust observer-based control strategy for n-DOF uncertain robot manipulators with fixed-time stability is developed. Firstly, the novel FxNTSM surface is proposed to quickly obtain a fixed-time convergence of the control errors without singularity. Secondly, to proactively deal with uncertain terms and exterior disturbances, the FxDO is developed based on a URED. The designed FxDO accurately approximates uncertain terms within a fixed time and contributes to significant chattering reduction in the traditional SMC. Finally, a robust observer-based control strategy is formed according to a combination of the FxNTSMC method and the FxDO, to offer global fixed-time stability for n-DOF uncertain robot manipulators.

### 3.1. Proposal of the FxNTSM Surface

To attain the fixed-time convergence of the control errors in system (3) without singularity, the novel FxNTSM surface was developed as:

(11) $$s=\varsigma +\Gamma e_{2}^{\gamma },$$

where $e_{2}^{\gamma }={\left[\begin{array}{ccc} e_{21}^{\gamma }, & \ldots , & e_{2n}^{\gamma } \end{array}\right]}^{T}$, $\Gamma =\mathrm{diag}\left(\begin{array}{ccc} \Gamma_{1}, & \ldots , & \Gamma_{n} \end{array}\right),\Gamma_{i}>0$, and $\varsigma ={\left[\begin{array}{ccc} {\left(1+e_{11}^{2}\right)}^{\gamma }\arctan\left(e_{11}\right), & \ldots & \left({\left(1+e_{1n}^{2}\right)}^{\gamma }\arctan\left(e_{1n}\right)\right) \end{array}\right]}^{T}$. γ is a number that can change according to the following relation:

(12) $$\gamma =0.5\left(\frac{\eta }{q}+\frac{q}{\eta }\right)+0.5\left(-\frac{\eta }{q}+\frac{q}{\eta }\right)\mathrm{sign}\left(\left|e_{1i}\right|-1\right).$$

It is noted that $\gamma =\{\begin{array}{c} \frac{q}{\eta }\;\left|e_{1i}\right|>1\;\\ \frac{\eta }{q}\;\left|e_{1i}\right|\leq 1 \end{array}$ in which η and q are positive odd integers and they are chosen along with the condition $1<\frac{\eta }{q}<2$, hence, $e_{2}^{\gamma }\in R\;\forall e_{2}\in R$. This precludes the generation of complex values. As a result, the proposed sliding surface has no singularity.

**Theorem** **1.** *Applying the novel FxNTSM surface in Equation (11), the trajectories of the control errors*$e_{1i},\;\left(i=1,\cdots ,n\right)$* will be approached to zero in fixed time*$\;t_{s}$.

**Proof** **of** **Theorem** **1.** Once the sliding motion of the proposed FxNTSM surface in Equation (11) occurs and satisfies the condition $s_{i}=0,\;\left(i=1,\cdots ,n\right)$ , a set of the following differential equations is also attained:

(13) $${\left(1+e_{1i}^{2}\right)}^{\gamma }\arctan\left(e_{1i}\right)+\Gamma_{i}e_{2i}^{\gamma }=0,\;i=1,\ldots ,n.$$

For i=1,…,n, Equation (13) can be rewritten as:

(14) $$\left(1+e_{1i}^{2}\right)\arctan{\left(e_{1i}\right)}^{\frac{1}{\gamma }}=-\Gamma_{i}^{\frac{1}{\gamma }}{\dot{e}}_{1i}.$$

Let $x=\arctan\left(e_{1i}\right)$, hence, the derivative of x is:

(15) $$\dot{x}={\left(1+e_{1i}^{2}\right)}^{-1}{\dot{e}}_{1i}.$$

With ${\dot{e}}_{1i}=\left(1+e_{1i}^{2}\right)\dot{x}$, Equation (14) yield:

(16) $$x^{\frac{1}{\gamma }}+\Gamma_{i}^{\frac{1}{\gamma }}\dot{x}=0.$$

The below two cases are considered.

The first case: $\left|e_{1i}\right|>1\to \gamma =\frac{q}{\eta }$: The initial starting point of the system’s trajectories is set far from the equilibrium point $\left|e_{1i}\right|>1$, Equation (16) becomes:

(17) $$\dot{x}=-\Gamma_{i}^{-\frac{\eta }{q}}x^{\frac{\eta }{q}}.$$

Lyapunov function ${ℒ}_{1}=0.5x^{2}$ is considered. The first-order time derivative of Lyapunov function, ${ℒ}_{1}$, is now calculated along with the obtained result in Equation (17) as:

(18) $${\dot{ℒ}}_{1}=x\dot{x}\;=-\Gamma_{i}^{-\frac{\eta }{q}}x^{\frac{\eta }{q}+1}.$$

Obviously, ${ℒ}_{1}>0$ and ${\dot{ℒ}}_{1}<0$. As a result, x and $\dot{x}$ asymptotically stabilize to the equilibrium point.

In stage $e_{1i}\left(0\right)\to \left|e_{1i}\right|=1$ then, $x\left(0\right)\to \left|x\right|=\frac{\pi }{4}$. Hence, the sliding motion takes place in the following computation time:

(19) $$\begin{array}{l} \int_{0}^{t_{1s_{i}}}dt=\Gamma_{i}^{\frac{\eta }{q}}\int_{\frac{\pi }{4}}^{x\left(0\right)}x^{\frac{-\eta }{q}}d\left(\left|x\right|\right)\\ t_{1s_{i}}=\Gamma_{i}^{\frac{\eta }{q}}\int_{\frac{\pi }{4}}^{x\left(0\right)}x^{\frac{-\eta }{q}}d\left(\left|x\right|\right)\\ ={\left[\Gamma_{i}^{\frac{\eta }{q}}\frac{q}{q-\eta }{\left(\left|x-\frac{\pi }{4}\right|\right)}^{1-\frac{\eta }{q}}\right]}_{\frac{\pi }{4}}^{x\left(0\right)}\\ \leq \Gamma_{i}^{\frac{\eta }{q}}\frac{q}{q-\eta }\left({\left(\frac{\pi }{2}\right)}^{1-\frac{\eta }{q}}-{\left|\frac{\pi }{4}\right|}^{1-\frac{\eta }{q}}\right). \end{array}$$

The second case: $\left|e_{1i}\right|\leq 1\to \gamma =\frac{\eta }{q}$: The initial starting point of the system’s paths is near the designated path $\left|e_{1i}\right|\leq 1$, so Equation (16) is written as:

(20) $$\dot{x}=-\Gamma_{i}^{-\frac{q}{\eta }}x^{\frac{q}{\eta }}.$$

Selecting Lyapunov function ${ℒ}_{2}=0.5x^{2}$, the first-order derivative of ${ℒ}_{2}$ according to time combining with Equation (20) results:

(21) $$\begin{array}{ll} {\dot{ℒ}}_{2} & =x\left(-\Gamma_{i}^{-\frac{q}{\eta }}x^{\frac{q}{\eta }}\right)\\ & =-\Gamma_{i}^{-\frac{q}{\eta }}x^{\frac{q}{\eta }+1}\\ & =-{\sqrt{2}}^{\frac{q}{\eta }+1}\Gamma_{i}^{-\frac{q}{\eta }}{ℒ}_{2}^{\frac{\left(\frac{q}{\eta }+1\right)}{2}}. \end{array}$$

The term $\frac{\eta }{q}$ is chosen along with the condition $1<\frac{\eta }{q}<2$/ As a result, $\frac{\left(\frac{q}{\eta }+1\right)}{2}\in \left(\frac{3}{4},1\right)$. With the statement presented in Lemma 1, we conclude that x can reach origin in finite-time. Due to $x=\arctan(e_{1i})$, $e_{1i}$ can approach zero in finite-time with computation time below:

(22) $$t_{2s_{i}}\leq \frac{\Gamma_{i}^{\frac{q}{\eta }}}{\left(1-\frac{q}{\eta }\right)}{\left|\arctan\left(e_{1i}\right)\right|}^{1-\frac{q}{\eta }}\leq \frac{\Gamma_{i}^{\frac{q}{\eta }}}{\left(1-\frac{q}{\eta }\right)}{\left(\frac{\pi }{2}\right)}^{1-\frac{q}{\eta }}.$$

From Equations (19) and (21), the convergence time that occurs in sliding motion is given below:

(23) $$t_{s}=\underset{1\leq i\leq n}{\max}\left\{t_{si}\right\}\leq \underset{1\leq i\leq n}{\max}\left\{t_{1si}+t_{2si}\right\}.$$

The value $t_{s}$ stated in Equation (23) only relates to the design constants. Consequently, the control errors $e_{1i}$ will surely attain the equilibrium point in fixed time. This proof is completed. □

### 3.2. Design of a Fixed-Time Disturbace Observer

The lumped uncertainty is approximated by an FxDO. This observer is designed based on a URED, as follows:

(24) $$\{\begin{array}{l} \delta_{0}={\hat{v}}_{2}-v_{2}\\ {\dot{\hat{v}}}_{2}=Z\left(v\right)u+A\left(v\right)+\hat{\delta }-\kappa_{1}\psi_{1}\left(\delta_{0}\right)\\ \dot{\hat{\delta }}=-\kappa_{2}\psi_{2}\left(\delta_{0}\right) \end{array},$$

where ${\hat{v}}_{2}$ indicates an approximated value of $v_{2}$, and $\kappa_{1}$ and $\kappa_{2}$ are observer gains. The terms $\psi_{1}\left(\delta_{0}\right)$ and $\psi_{2}\left(\delta_{0}\right)$ are designed based on URED in [39], as follows:

(25) $$\{\begin{array}{l} \psi_{1}\left(\delta_{0}\right)={\left|\delta_{0}\right|}^{\frac{1}{2}}\mathrm{sign}\left(\delta_{0}\right)+\varpi {\left|\delta_{0}\right|}^{\frac{3}{2}}\mathrm{sign}\left(\delta_{0}\right)\\ \psi_{2}\left(\delta_{0}\right)=\frac{1}{2}\mathrm{sign}\left(\delta_{0}\right)+2\varpi \delta_{0}+\frac{3}{2}\varpi^{2}{\left|\delta_{0}\right|}^{2}\mathrm{sign}\left(\delta_{0}\right) \end{array}.$$

**Theorem** **2.** * Applying the proposed observer in Equation (24), when the term* $\left|{\dot{\delta }}_{i}\left(v,\Delta ,\tau_{d}\right)\right|<{\bar{\varrho }}_{i}$ *in Assumption 2 is satisfied, then the estimation error of the proposed observer will converge to zero in fixed time, independent of the initial condition and exterior disturbances.*

**Proof** **of** **Theorem** **2.** Differentiating the first order of $\delta_{0}$ , one obtains:

(26) $$\begin{array}{ll} {\dot{\delta }}_{0} & ={\dot{\hat{v}}}_{2}-{\dot{v}}_{2}\\ & =\hat{\delta }-\delta -\kappa_{1}\psi_{1}\left(\delta_{0}\right)\\ & =\delta_{1}-\kappa_{1}\psi_{1}\left(\delta_{0}\right), \end{array}$$

where $\delta_{1}=\hat{\delta }-\delta$ represents the estimation error and $\delta_{1}={\left[\begin{array}{ccc} \delta_{11}, & \cdots , & \delta_{1n} \end{array}\right]}^{T}$ is limited by a known constant $\left|\delta_{1i}\right|\leq ℬ,\;i=1,\cdots ,n$.

Taking the first-order time derivative of $\delta_{1}$ and referring to Equation (24), we can gain:

(27) $$\begin{array}{ll} {\dot{\delta }}_{1} & =\dot{\hat{\delta }}-\dot{\delta }\\ & =-\kappa_{2}\psi_{2}\left(\delta_{0}\right)-\dot{\delta }. \end{array}$$

The observer gains $\kappa_{1}$ and $\kappa_{2}$ are selected in the set, as follows:

(28) $$ℋ=\left\{\left(\kappa_{1},\kappa_{2}\right)\in R^{2}|0<\kappa_{1}\leq 2\sqrt{\lambda },\kappa_{2}>\frac{\kappa_{1}^{2}}{4}+\frac{4\lambda^{2}}{\kappa_{1}^{2}}\right\}\cup \left\{\left(\kappa_{1},\kappa_{2}\right)\in R^{2}|\kappa_{1}>2\sqrt{\lambda },\kappa_{2}>2\lambda \right\},$$

in which $\lambda =\max\left\{\begin{array}{cccc} {\bar{\varrho }}_{1}, & {\bar{\varrho }}_{2}, & \cdots , & {\bar{\varrho }}_{n} \end{array}\right\}$.

Referring to Equation (3) in the study [39], it is seen that Equations (26) and (27) are given the same form. In addition, the estimated value $\dot{\delta }$ is bounded by Assumption 2 and corresponds to ${\ddot{f}}_{0}$ in the study [39], as stated in Equation (3). As a result, the proposed observer will exactly estimate the lumped uncertainty when we can achieve $\delta_{1i}=0,\;\left(i=1,\cdots ,n\right)$ within the fixed time, $T_{0}$ as observe in [39], and the convergence time of the proposed observer is calculated by assigning $\kappa_{1},\kappa_{2}$ , and ϖ for any initial conditions (readers can refer to Equations (5)–(9) and Appendix A in [39]). Consequently, we can conclude that using the proposed observer in Equation (24) with the suitable conditions, we can exactly estimate the lumped uncertainty in fixed-time, independent of the initial condition, and despite disturbances. This proof is completed. □

### 3.3. Design of a FxNTSMC Method

Computing the first-order derivative of FxNTSM surface according to time, we gain:

(29) $$\dot{s}=\dot{\varsigma }+\gamma \Gamma \mathrm{diag}\left(\begin{array}{ccc} e_{21}^{\gamma -1}, & \ldots , & e_{2n}^{\gamma -1} \end{array}\right){\dot{e}}_{2}\;\;=\dot{\varsigma }+\gamma \Gamma \mathrm{diag}\left(\begin{array}{ccc} e_{21}^{\gamma -1}, & \ldots , & e_{2n}^{\gamma -1} \end{array}\right)\left(Ζ\left(v\right)u+ℋ\left(v\right)+\delta \left(v,\Delta ,\tau_{d}\right)\right)\;\;=\dot{\varsigma }+\gamma \Phi \left(Ζ\left(v\right)u+ℋ\left(v\right)+\delta \left(v,\Delta ,\tau_{d}\right)\right),$$

where $\Phi =\mathrm{diag}\left(\Phi_{1},\cdots ,\Phi_{n}\right)=\Gamma \mathrm{diag}\left(\begin{array}{ccc} e_{21}^{\gamma -1}, & \ldots , & e_{2n}^{\gamma -1} \end{array}\right)$.

Then, the control torques of FxNTSMC are designed based on the proposed FxNTSM Surface in Section 3.1 and the proposed FxDO in Section 3.2, as follows:

(30) $$u=-{Ζ}^{-1}\left(v\right)\left(ℋ\left(v\right)+ℱ+\hat{\delta }\right)-{Ζ}^{-1}\left(v\right)\left(K{\left|s\right|}^{\beta }\mathrm{sign}\left(s\right)+ℬ\mathrm{sign}\left(\Phi s\right)\right),$$

where β>2,

$$ℱ={\left[\begin{array}{ccc} \frac{{\left(1+e_{11}^{2}\right)}^{\gamma -1}}{\Gamma_{1}\gamma }\left(1+2\gamma e_{11}\arctan\left(e_{11}\right)\right)e_{21}^{2-\gamma }, & \ldots , & \frac{{\left(1+e_{1n}^{2}\right)}^{\gamma -1}}{\Gamma_{n}\gamma }\left(1+2\gamma e_{1n}\arctan\left(e_{1n}\right)\right)e_{2n}^{2-\gamma } \end{array}\right]}^{T}.$$

**Theorem** **3.**  *If the control torques of FxNTSMC are designed for robot manipulators (1) based on the proposed FxNTSM Surface in Equation (11) and the proposed FxDO in Equation (24) which is given in Equation (30) then the proposed controller* *offers global fixed-time stability for robot manipulators.*

**Proof** **of** **Theorem** **3.** Inserting the control input (30) into Equation (29) obtains

(31) $$\dot{s}=\gamma \Phi \left(-K{\left|s\right|}^{\beta }\mathrm{sign}\left(s\right)-ℬ\mathrm{sign}\left(s\right)-\delta_{1}\right).$$

A set of the differential equations from Equation (31) is described as:

(32) $${\dot{s}}_{i}=\gamma \Phi_{i}\left(-K{\left|s_{i}\right|}^{\beta }\mathrm{sign}\left(s_{i}\right)-ℬ\mathrm{sign}\left(s_{i}\right)-\delta_{1i}\right),\;i=1,\cdots ,n.$$

The Lyapunov function candidate is defined as ${ℒ}_{3i}=0.5s_{i}^{2}\;i=1,2,\ldots ,n$. Then, differentiating Lyapunov function gives:

(33) $$\begin{array}{ll} {\dot{ℒ}}_{3i} & =s_{i}{\dot{s}}_{i}\\ & =s_{i}\gamma \Phi_{i}\left(-K{\left|s_{i}\right|}^{\beta }\mathrm{sign}\left(s_{i}\right)-ℬ\mathrm{sign}\left(s_{i}\right)-\delta_{1i}\right)\\ & =\gamma \left(-K\Phi_{i}{\left|s_{i}\right|}^{\beta +1}-ℬ\Phi_{i}\left|s_{i}\right|-\Phi_{i}s_{i}\delta_{1i}\right)\\ & \leq -\gamma K\Phi_{i}{\left|s_{i}\right|}^{\beta +1}-\gamma \left(ℬ-\left|\delta_{1i}\right|\right)\Phi_{i}\left|s_{i}\right|\\ & \leq -\gamma K\Phi_{i}{\left|s_{i}\right|}^{\beta +1}-\mu \left|s_{i}\right|\leq 0, \end{array}$$

where $\mu =\gamma \left(ℬ-\left|\delta_{1i}\right|\right)\Phi_{i}>0$.

The suggested FxNTSM surface will be attained the equilibrium point in finite-time $t_{r}$. It means that the convergence and stability of the designed control strategy are guaranteed in finite time $t=t_{r}+t_{s}$.

We will prove that Equation (33) is fixed-time stable. Therefore, Equation (33) is rewritten as follows:

(34) $${\dot{ℒ}}_{3i}\leq -2^{\frac{\beta +1}{2}}\gamma K\Phi_{i}{ℒ}_{3i}^{\frac{\beta +1}{2}}-2^{\frac{1}{2}}\mu {ℒ}_{3i}^{\frac{1}{2}}=-Z_{1}{ℒ}_{3i}^{\frac{\beta +1}{2}}-Z_{2}{ℒ}_{3i}^{\frac{1}{2}},$$

where $Z_{1}=2^{\frac{\beta +1}{2}}\gamma K\Phi_{i}>0$ , $Z_{2}=2^{\frac{1}{2}}\mu >0$.

Due to $Z_{1},Z_{2}>0$ , and $\frac{\beta +1}{2}>1$ , based on Lemma 2, the convergence time of the reaching phase is bounded by

(35) $$t_{ri}\leq \frac{2}{Z_{1}}\frac{1}{\beta -1}+\frac{2}{Z_{2}}.$$

From Equations (23) and (35), it can be concluded that the proposed control system can also obtain convergence and stability within the following fixed time:

(36) $$t=t_{r}+t_{s}=\underset{1\leq i\leq n}{\max}\left\{t_{ri}\right\}+\underset{1\leq i\leq n}{\max}\left\{t_{1si}+t_{2si}\right\}.$$

This proof is completed. □

**Figure 1:**
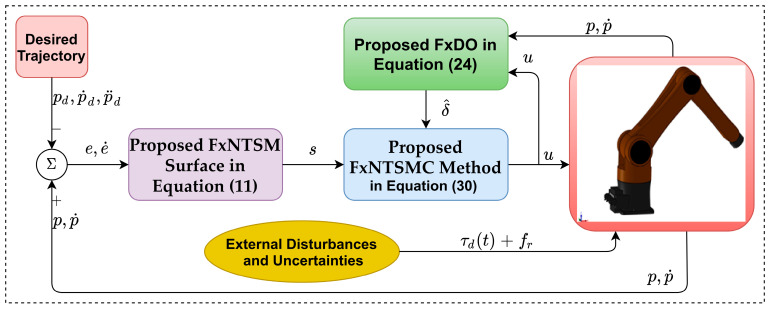
Block diagram of the proposed control system.

The design procedure of the proposed controller is briefly explained in Figure 1.

## 4. Illustrative Example

**Figure 2:**
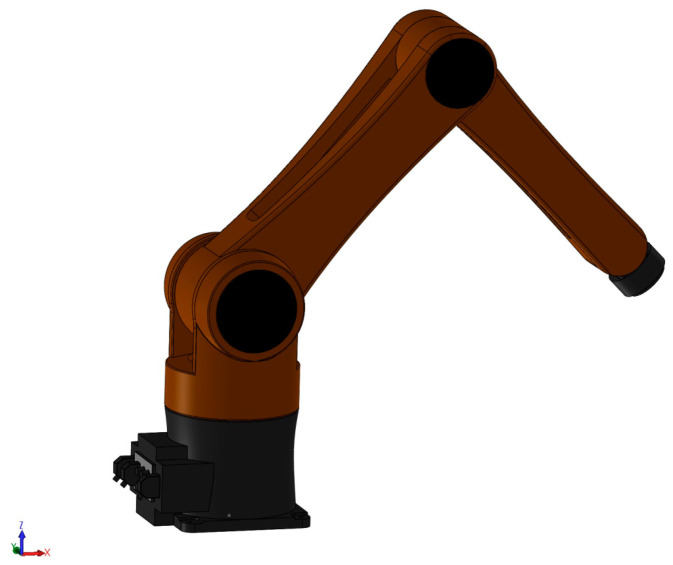
3D Description of a 3-DOF manipulator based on SOLIDWORKS.

Figure 2 shows a 3D Description of a 3-DOF manipulator based on SOLIDWORKS.

The kinematic design and dynamic computation of the robot system were conducted based on the PUMA 560 manipulator [44,45]. To facilitate the presentation of simulation performance, the manipulator is designed with three degrees of freedom (DOF). In this paper, SOLIDWORKS was used to design the robot manipulator parts, the structure of the robot, the addition of the coordinate system, the measuring devices, and the direction of gravitational force. Each mechanical component of the robot system was constructed separately and assembled using suitable joints. Using the Simscape Multibody Link Tool from SOLIDWORKS, we created two types of files. The XML file included important parameters of the robot’s mechanical components and parameters of the coordinate system of the assembly environment, such as the center of mass, length of link, mass, inertia moment, etc. The detailed design parameters of the robot can be found in Table 2. The STEP files comprised the 3-D computer-aided design (CAD) model of the mechanical parts. To achieve a realistic model when performing simulations, both file types were included in the MATLAB/Simulink environment via Simscape Multibody Link. Furthermore, the lumped uncertainty, including uncertain dynamics, exterior disturbances, and friction forces was assumed to add to the robot manipulator. The mechanical model of the robot in SOLIDWORKS was the same as the real robot model. In addition, the simulated environment of the robot was considered to be the same as the real conditions. Therefore, it was determined that the SOLIDWORKS model of the robot was able to be used to validate the control performance effectively.

As we know, there exists a discrepancy between the real model and the calculated model. To simulate these model errors, we included $\Delta M\left(p\right),\Delta C\left(p,\dot{p}\right),\;\Delta G\left(p\right)$. Throughout the simulation, the unidentified dynamics were assumed to be ΔM(p)=0.2M(p), $\Delta C\left(p,\dot{p}\right)=0.2C\left(p,\dot{p}\right)$, and ΔG(p)=0.2G(p).

To test the robustness of the proposed control strategy, the friction force and exterior disturbance were assumed at each joint as $f_{r1}\left(\dot{p}\right)+\tau_{d1}\left(t\right)=0.1\mathrm{sign}\left({\dot{p}}_{1}\right)+2{\dot{p}}_{1}+2.5\sin(10t-20){\dot{p}}_{1}-2.2p_{1}^{3}\;\left(N\cdot m\right)$, $f_{r2}\left(\dot{p}\right)+\tau_{d2}\left(t\right)=0.1\mathrm{sign}\left({\dot{p}}_{2}\right)+2{\dot{p}}_{2}+2.3\sin(10t-20){\dot{p}}_{2}-4.2p_{2}^{3}\;\left(N\cdot m\right)$, and $f_{r3}\left(\dot{p}\right)+\tau_{d3}\left(t\right)=0.1\mathrm{sign}\left({\dot{p}}_{3}\right)+2{\dot{p}}_{3}+3.5\sin(10t-20){\dot{p}}_{3}+3.2p_{3}^{3}\;\left(N\cdot m\right)$.

To evaluate the motion control of the robotic manipulator when approaching and maintaining a specified path, the configuration of the trajectory was designed in the form of a circle in XYZ coordinate system, as follows: X=0.85−0.01t (m), Y=0.2+0.2sin(0.5t) (m), Z=0.7+0.2cos(0.5t) (m), and t≤20 s. The selected reference trajectory was a circle in three-dimensional spaces. This meant that the amplitude in the YZ direction of this reference trajectory changed over time for a given periodicity, and the amplitude in the X direction of this reference trajectory changed linearly over time. Therefore, it served as a general trajectory for verifying tracking control. In addition, to check aspects of any initial conditions ($\left|e_{1i}\right|>1$ and $\left|e_{1i}\right|\leq 1$), the manipulator was configured with the initial starting points at each joint as: $p_{1}=-1.6\;\left(\mathrm{rad}\right)$, $p_{2}=-1\;\left(\mathrm{rad}\right)$, and $p_{3}=-0.5\;\left(\mathrm{rad}\right)$.

In comparison, other state-of-the-art controllers, including NFTSMC1, based on the method of [46], and NFTSMC2, based on the method of [47], have been considered to compare with the proposed controller in aspects such as: convergence rate, chattering, robustness to cope with uncertain terms, and accuracy in tracking control.

The control torques of NFTSMC1 were constructed for the manipulator as:

(37) $$\{\begin{array}{l} s=\dot{e}+\varphi_{1}e+\phi_{1}{\left|e\right|}^{\omega_{1}}\mathrm{sign}\left(e\right)\\ u=-{Ζ}^{-1}\left(v\right)\left(ℋ\left(v\right)+\left(\varphi_{1}+\phi_{1}\omega_{1}{\left|e\right|}^{\omega_{1}-1}\right)\dot{e}+K_{1}s+\left(\bar{\varrho }+ℬ\right)\mathrm{sign}\left(s\right)\right) \end{array},$$

where $\varphi_{1},\phi_{1},K_{1}$ are the design positive constants, $0<\omega_{1}<1$.

The control torques of NFTSMC2 were designed for the manipulator as:

(38) $$\{\begin{array}{l} s=\dot{e}+\frac{2\varphi_{2}}{1+{℮}^{-\theta_{2}\left(\left|e_{i}\right|-\varepsilon_{2}\right)}}e+\frac{2\phi_{2}}{1+{℮}^{\sigma_{2}}\left(\left|e_{i}\right|-\varepsilon_{2}\right)}{\left|e\right|}^{\omega_{2}}\mathrm{sign}\left(e\right)\\ u=-Z^{-1}\left(v\right)\left(\begin{array}{c} H\left(v\right)+\frac{2\varphi_{2}}{1+{℮}^{-\theta_{2}}\left(\left|e_{i}\right|-\varepsilon_{2}\right)}\dot{e}+\frac{2\varphi_{2}\theta_{2}\dot{e}\mathrm{sign}\left(e\right){℮}^{-\theta_{2}\left(\left|e_{i}\right|-\varepsilon_{2}\right)}}{{\left(1+{℮}^{-\theta_{2}\left(\left|e_{i}\right|-\varepsilon_{2}\right)}\right)}^{2}}e+\frac{2\phi_{2}\omega_{2}}{1+{℮}^{\sigma_{2}\left(\left|e_{i}\right|-\varepsilon_{2}\right)}}{\left|e\right|}^{\omega_{2}-1}\dot{e}\\ -\frac{2\phi_{2}\sigma_{2}\dot{e}{℮}^{\sigma_{2}\left(\left|e_{i}\right|-\varepsilon_{2}\right)}}{{\left(1+{℮}^{\sigma_{2}\left(\left|e_{i}\right|-\varepsilon_{2}\right)}\right)}^{2}}{\left|e\right|}^{\omega_{2}}+K_{2}s+\left(\bar{\varrho }+B\right)\mathrm{sign}\left(s\right) \end{array}\right) \end{array}$$

where $\varphi_{2},\phi_{2},\theta_{2},\sigma_{2},K_{2}$ are the design positive constants, $0<\omega_{2}<1$, $\varepsilon_{2}={\left(\frac{\phi_{2}}{\varphi_{2}}\right)}^{\frac{1}{\left(1-\omega_{2}\right)}}$.

Three control systems were applied to stabilize the manipulator (1); their control parameters are presented in Table 3. The proposed FxNTSMC was developed based on the proposed FxDO, hence, the FxDO parameters are also reported in Table 3.

For the convenience of accuracy comparison, the root-mean-square errors were calculated from the 2nd sec to the 20th s, as described in Table 4.

**Remark** **1.** *For an optimal choice of control parameters, while guaranteeing fairness between control strategies, several choice methods were applied to attain good tracking performance for all three control strategies in the aspects of fast convergence speed, high tracking precision, stability, and chattering reduction. The control parameter selection of the proposed sliding surface ensured the conditions presented below Equations (11) and (12) attained the fixed-time convergence of the control errors in system (3) without singularity. The observer gains* $\kappa_{1}$ *and* $\kappa_{2}$ *were selected in the set, as stated in Equation (28). The formula* $\left(\bar{\varrho }+ℬ\right)$ *is the sliding gain of the reaching control law in Equations (37) and (38). These parameters are assigned a value greater than the upper-bound value of the lumped unknown uncertainty. Therefore, this condition guarantees asymptotic stabilization for the control system. With the selection of other control parameters of all three controllers, the reader can easily find instructions or conditions presented below the equations of the control signal. Furthermore, the selection of control parameters is performed by repeated experiments to get the optimal control parameters.*

**Figure 3:**
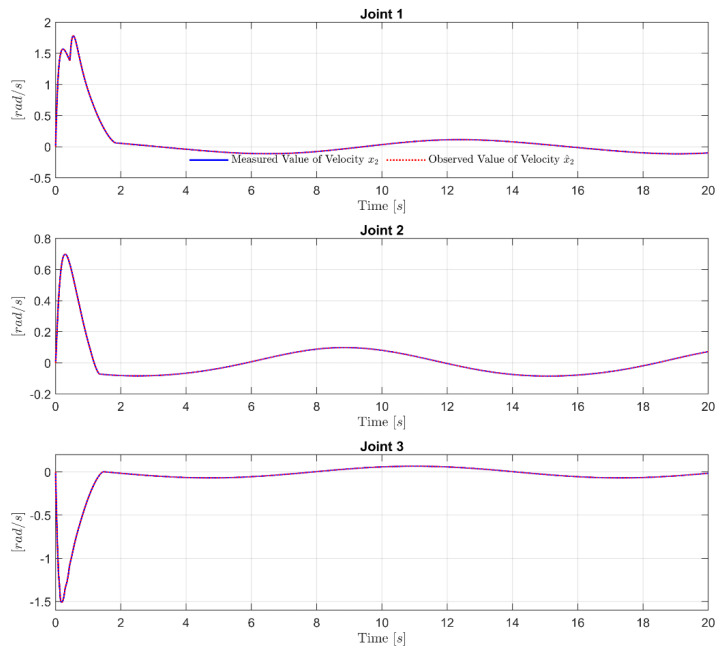
Measured value of velocity $x_{2}$ and observed value of velocity ${\hat{x}}_{2}$ at each Joint.

**Figure 4:**
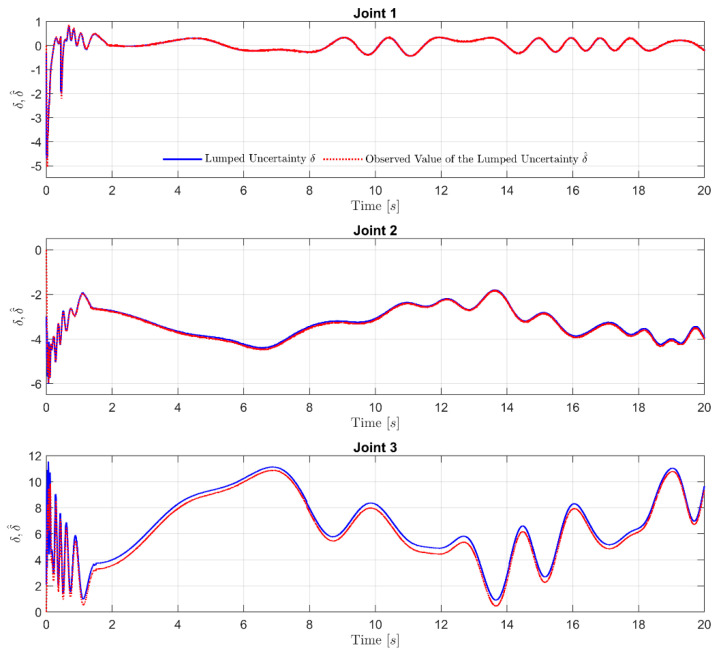
Supposed value of the lumped uncertainty and observed value of the lumped uncertainty at each Joint.

The effectiveness of the proposed FxDO is firstly considered in order to evaluate its approximation capability. As shown in Figure 3, the trajectory of the observed velocity completely coincided with the trajectory of the measured velocity from the sensor at the initial time, and remained until the end of the simulation time. In Figure 4, we note that the proposed FxDO exactly approximated the supposed value of the lumped uncertainty at each joint in two aspects: amplitude and frequency. The estimation errors of the proposed FxDO converged to zero within the fixed time. The convergence property of the observer in a fixed time is important for separation-like properties in the robot manipulator. It implies that the estimation errors of the observer reach zero before the real trajectories of the robot have flowed to infinity. Consequently, it provides timely and accurate information about the uncertain terms to the control system, and this plays a major role in enhancing robustness against uncertainty and reducing the dynamic computation burden.

**Figure 5:**
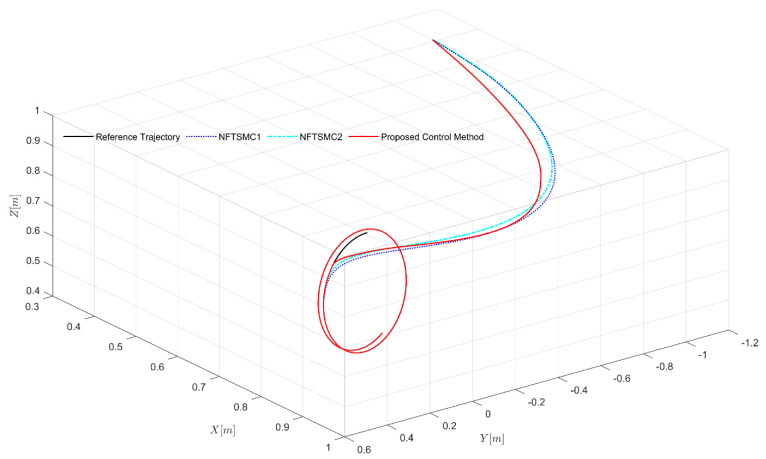
Specified path and actual path of the end effector of the robot under three control strategies in 3-dimensional space (XYZ).

**Figure 6:**
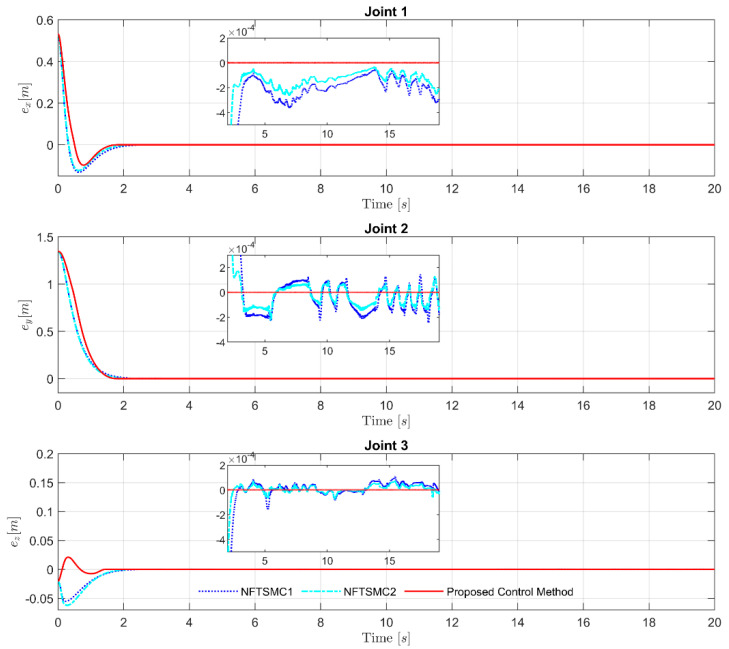
Path of the control errors in 3-dimensional space (XYZ).

**Figure 7:**
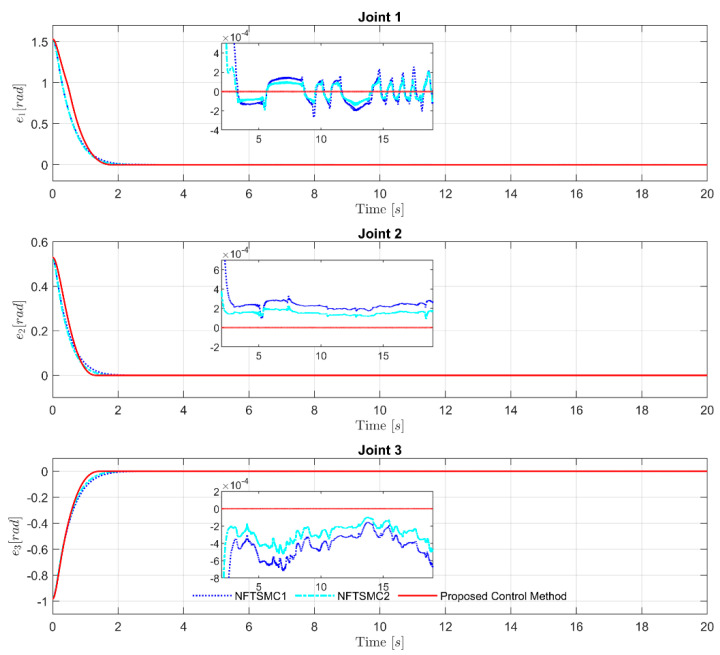
Path of the control errors at each joint.

The control performances of the three different control strategies for a 3-DOF un-certain robot manipulator are shown in Figure 5, Figure 6 and Figure 7. In Figure 5, it can be seen that the initial point of the end effector of the robot was designed far from the designated path for investigating fixed-time convergence with arbitrary initial conditions. The control performances in Figure 5 show that all three controllers guaranteed high tracking accuracy for the robot, while the actual path under the suggested control strategy reached the designated path with the greatest rapidity, due to dynamic coefficients designed in the FxNTSM surface that could be adjusted to the control errors, as stated in Section 3.1.

Performing a detailed comparison of control errors in Figure 6 and Figure 7, and using a quantification method for the root-mean-square errors, as reported in Table 4, it can be easily observed that NFTSMC2 offered better tracking accuracy than NFTSMC1. It is noteworthy that the suggested observer-based control algorithm with robust anti-uncertainty ability provided the highest tracking accuracy compared to the two remaining control strategies; it greatly improved the control performance with excellent accuracy and small overshoot. The control errors in the proposed observer-based controller converged fastest to the equilibrium point in fixed time.

**Figure 8:**
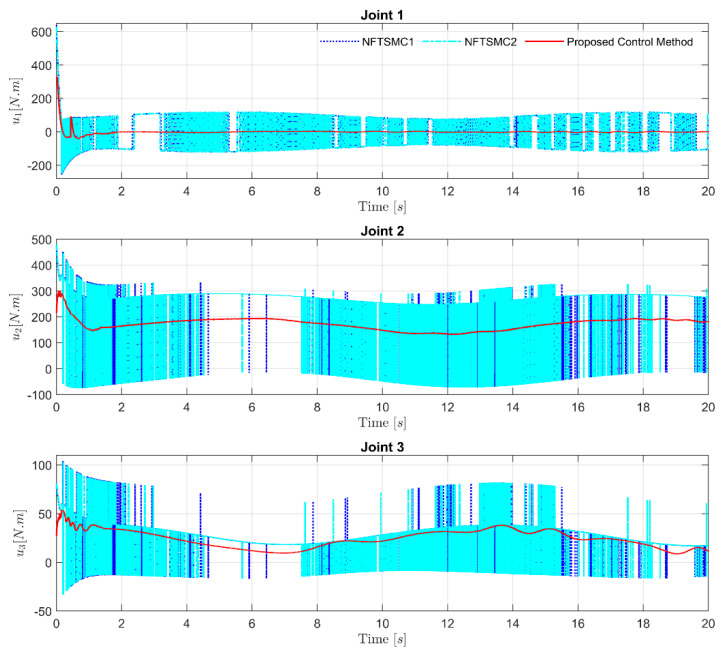
Control torque of the three different control strategies and comparison of chattering phenomena at each Joint.

NFTSMC1 and NFTSMC2 were accorded same the sliding value to cope with the effects of the lumped uncertainty. Therefore, both controllers provided nonsmooth control signals with high-frequency oscillation. Meanwhile, by feeding the information of the uncertain terms accurately to the control loop from the proposed FxDO, the performance of the controller was not only significantly improved, but the chattering phenomenon was also effectively reduced, as shown in Figure 8.

## 5. Conclusions

Our paper developed an observer-based control algorithm for n-DOF uncertain robot manipulators with important contributions as follows: The proposed FxNTSM surface guaranteed that it obtained fixed-time convergence of the control errors without singularity. The designed FxDO based on a URED accurately approximated uncertain terms within a fixed time, and contributed to a significant chattering reduction in the traditional SMC. In addition, the proposed FxDO removed the requirements for measuring acceleration. The proposed controller has a simple design suitable for application in actual robots. The design was formulated according to a combination of the FxNTSMC method and the designed FxDO to offer global fixed-time stability for robot manipulators. The convergence time was bounded, and it could be pre-computed by setting appropriate design constants.

Through the quality evaluation of the control performance and comparisons, the proposed controller obtained high tracking accuracy, small overshoot, chattering reduction, robust anti-uncertainty ability, and fast convergence of both the tracking errors and the estimation errors within fixed time. In addition, the proposed FxNTSMC was proven to obtain global stability in fixed time using the Lyapunov criteria.

Following this work, we plan to propose an FTC for robotic manipulators, which will consider faults in the measuring sensors. In addition, the proposed controller will also be applicable in real robot manipulators.

## Figures and Tables

**Table 1:** Notations and nomenclature.

Notation	Description
$R^{n}$	the real n-dimensional space
$R_{+}$	the set of positive real numbers
$R^{n\times m}$	the set of m by n real matrices
v	given vector or matrix
$\cdot^{T}$	the transpose of
|·|	absolute value of
‖·‖	Euclidean norm of
p	vector of joint angular position, $p\in R^{n\times 1}$
$\dot{p}$	vector of joint angular velocity, $\dot{p}\in R^{n\times 1}$
$\ddot{p}$	vector of joint angular acceleration, $\ddot{p}\in R^{n\times 1}$
M(p)	positive–definite and symmetric matrix of inertia parameters, $M\left(p\right)\in R^{n\times n}$
$\hat{M}\left(p\right)$	estimated part of M(p) $,\;\hat{M}\left(p\right)\in R^{n\times n}$
ΔM(p)	uncertain dynamic part of M(p) $,\;\Delta M\left(p\right)\in R^{n\times n}$
$C\left(p,\dot{p}\right)$	matrix of the Coriolis and centripetal forces, $C\left(p,\dot{p}\right)\in R^{n\times n}$
$\hat{C}\left(p,\dot{p}\right)$	estimated part of $C\left(p,\dot{p}\right)$ $,\;\hat{C}\left(p,\dot{p}\right)\in R^{n\times n}$
$\Delta C\left(p,\dot{p}\right)$	uncertain dynamic part of $C\left(p,\dot{p}\right)$ $,\;\Delta C\left(p,\dot{p}\right)\in R^{n\times n}$
G(p)	vectors of the gravitational force, $G\left(p\right)\in R^{n\times 1}$
$\hat{G}\left(p\right)$	estimated part of G(p) $,\;\hat{G}\left(p\right)\in R^{n\times 1}$
ΔG(p)	uncertain dynamic part of G(p) $,\;\Delta G\left(p\right)\in R^{n\times 1}$
$f_{r}\left(\dot{p}\right)$	vectors of the friction force, $f_{r}\left(\dot{p}\right)\in R^{n\times 1}$
τ	vector of the control input torque, $\tau \in R^{n\times 1}$
$\tau_{d}\left(t\right)$	unknown time-varying external disturbance, $\tau_{d}\left(t\right)\in R^{n\times 1}$
A(v)	lumped nominal part of the robot
Z(v)	a smooth function
$\delta \left(v,\Delta ,\tau_{d}\right)$	lumped unknown uncertainty
$p_{d},{\dot{p}}_{d},{\ddot{p}}_{d}$	desired trajectory, the first and second derivative of $p_{d}$ under varying time, $p_{d},{\dot{p}}_{d},{\ddot{p}}_{d}\in R^{n\times 1}$
e	vector of control errors, $e={\left[\begin{array}{cc} e_{1}^{T} & e_{2}^{T} \end{array}\right]}^{T}\in R^{2n}$
$e_{1},\;e_{2},{\dot{e}}_{2}$	control errors, the first and second derivative of $e_{1}$ under time-varying, $e_{1}={\left[\begin{array}{ccc} e_{11}, & \ldots , & e_{1n} \end{array}\right]}^{T},\;e_{2}={\left[\begin{array}{ccc} e_{21}, & \ldots , & e_{2n} \end{array}\right]}^{T},{\dot{e}}_{2}\in R^{n\times 1}$. $e_{2}$ is the time derivative of $e_{1}$
s	vector of FxNTSM surface, $s\in R^{n\times 1}$
$\varrho_{i},{\bar{\varrho }}_{i},\kappa_{1},\kappa_{2},\sigma ,K$	positive constants
℮	Euler’s number
RMSE(·)	Root-mean-square error

**Table 2:** The detailed design parameters of the robot.

Description	Symbol	Value	Unit
Mass of each link	$m_{1}$	33.429	kg
	$m_{2}$	34.129	kg
	$m_{3}$	15.612	kg
Length of link	$l_{1}$	250	mm
	$l_{2}$	700	mm
	$l_{3}$	600	mm
Center of mass	${\left[l_{c1x},l_{c1y},l_{c1z}\right]}^{T}$	${\left[0,0,-74.610\right]}^{T}$	mm
	${\left[l_{c2x},l_{c2y},l_{c2z}\right]}^{T}$	${\left[347.7,0,0\right]}^{T}$	mm
	${\left[l_{c3x},l_{c3y},l_{c3z}\right]}^{T}$	${\left[314.2,0,0\right]}^{T}$	mm
Inertia	${\left[I_{1xx},I_{1yy},I_{1zz}\right]}^{T}$	${\left[0.7486,0.5518,0.5570\right]}^{T}$	kg·m^2^
	${\left[I_{2xx},I_{2yy},I_{2zz}\right]}^{T}$	${\left[0.3080,2.4655,2.3938\right]}^{T}$	kg·m^2^
	${\left[I_{3xx},I_{3yy},I_{3zz}\right]}^{T}$	${\left[0.0446,0.7092,0.7207\right]}^{T}$	kg·m^2^

**Table 3:** Design parameters of the three control systems.

Control Method	Control Parameter
NFTSMC1	$\varphi_{1}=5,\;\phi_{1}=5,\;\omega_{1}=0.8,\;K_{1}=5,\;\bar{\varrho }=13,\;ℬ=0.1$
NFTSMC2	$\varphi_{2}=5,\;\phi_{2}=5,\;\theta_{2}=0.9,\;\sigma_{2}=1.2$
	$\omega_{2}=0.8,\;K_{2}=5,\;\bar{\varrho }=13,\;B=0.1$
Proposed FxNTSMC	q=3, η=5, Γ=diag(0.4,0.4,0.4), K=5, ℬ=0.1
	$\kappa_{1}=\mathrm{diag}\left(18,18,18\right),\;\kappa_{2}=\mathrm{diag}\left(180,180,180\right),\;\varpi =\mathrm{diag}\left(2,2,2\right)$

**Table 4:** Root-mean-square errors.

Control Method	Root-Mean-Square Errors from the 2nd s to the 20th s					
	$RMSE\left(e_{x}\right)$	$RMSE\left(e_{y}\right)$	$RMSE\left(e_{z}\right)$	$RMSE\left(e_{1}\right)$	$RMSE\left(e_{2}\right)$	$RMSE\left(e_{3}\right)$
NFTSMC1	0.6241	$0.9\times 10^{-3}$	$0.1\times 10^{-3}$	$0.11\times 10^{-2}$	$0.3\times 10^{-3}$	$0.7\times 10^{-3}$
NFTSMC2	0.6240	$0.3\times 10^{-3}$	$0.1\times 10^{-3}$	$0.3744\times 10^{-3}$	$0.1582\times 10^{-3}$	$0.3310\times 10^{-3}$
Proposed FxNTSMC	$0.0753\times 10^{-7}$	$0.1363\times 10^{-7}$	$0.1553\times 10^{-7}$	$0.1595\times 10^{-7}$	$0.1597\times 10^{-7}$	$0.1476\times 10^{-7}$

## Data Availability

The data sets generated and/or analyzed during the current study are available from the corresponding author on reasonable request.
